# Volatile Insensitivity and Localized Alkaloids Support Commensalism in the *Oxytropis sericea–Alternaria oxytropis* Association

**DOI:** 10.1007/s10886-026-01773-9

**Published:** 2026-10-21

**Authors:** Jackson R. Strand, Laura A. Burkle, Daniel Cook, Dale R. Gardner, Barbara Keith, Rosie Wallander, David K. Weaver, Tracy M. Sterling

**Affiliations:** 1 Department of Land Resources and Environmental Sciences, Montana State University, Bozeman, MT USA; 2 Department of Ecology, Montana State University, Bozeman, MT USA; 3 Research Plant Physiologist, USDA-ARS Poisonous Plant Research Laboratory, Logan, UT USA

**Keywords:** Fungal endophyte, Plant-fungal symbiosis, Pollinator attraction, Headspace GC-MS

## Abstract

The endophytic fungus *Alternaria oxytropis* is vertically transmitted through silky crazyweed (*Oxytropis sericea* Nutt.) and produces the neurotoxin swainsonine, yet its influence on host chemical signaling remains unclear. Here we characterize the floral and foliar volatile organic compound (VOC) profiles of endophyte-infected (E+) and endophyte-free (E-) *O. sericea* using dynamic headspace GC-MS on greenhouse- and field-grown plants – the first characterization of volatile profiles in this species. Endophyte infection had a negligible effect on VOC composition, explaining less than 1% of compositional variance in both greenhouse and field settings, with no compound differing consistently by infection status. Seven volatiles were reliably detected across both environments. The dominant axes of variation (PCA) instead reflected differences among individuals and between size-matched E+/E- plant across years. These accounted for most compositional variance (72.6% greenhouse, 64.9% field), and VOC profiles differed markedly between collection years, likely reflecting fine-scale environmental and physiological variation. Ancillary nectar assays and pollinator observations revealed no consistent differences between E + and E- plants, and seed set was unaffected regardless of endophyte status. Swainsonine in floral organs was roughly an order of magnitude lower than in leaves and near-trace in nectar, indicating that any localized chemical defense is likely decoupled from scent chemistry. Together, these findings position the *Oxytropis-Alternaria* association toward the commensal end of the symbiotic continuum: the fungus maintains vertical transmission while the host shows no detectable effect on volatile signaling, pollinator attraction, or reproductive output, despite the resources required to sustain fungal alkaloid biosynthesis. This system offers a tractable model for understanding how endophyte associations persist in the absence of measurable host benefit.

**Supplementary Information:**

The online version contains supplementary material available at https://doi.org/10.1007/s10886-026-01773-9.

## Introduction

Symbioses are often understood as exchanges: hosts invest resources, and in return, microbial partners provide protection, nutrients, or stress tolerance (Sørensen et al. 2019; Hawkes et al. 2020). This logic underpins the persistence of mutualism and explains the costs of parasitism, but some associations resist either label, including that between *Oxytropis sericea* Nutt. (silky crazyweed), a perennial legume of western North America, and its alkaloid-producing fungal endophyte *Alternaria oxytropis*. The endophyte produces swainsonine, an indolizidine alkaloid potent enough to cause severe livestock poisoning (Molyneux and James 1982; Cook et al. 2009b), and yet no consistent effect of infection on host survival, fecundity, or stress tolerance has been detected (Vallotton et al. 2012; Klypina et al. 2017; Harrison et al. 2021; Keith et al. 2025).

Fungal endophytes are symptomless symbionts that colonize plant tissues systemically and persist across host generations (Carroll 1988; Faeth 2002; Rodriguez et al. 2009). In grasses, they are canonical mutualists: endophyte-produced alkaloids deter insect and mammalian herbivores and enhance tolerance to drought and heat, with measurable fitness gains for infected hosts (Shankar Naik 2019; Fu et al. 2024). The *Oxytropis*-*Alternaria* association departs from this pattern on both fronts. Swainsonine accumulates systemically in *O. sericea* (Ralphs et al. 2011) but does not function defensively. Specialist insects consume infected and uninfected plants alike (Thompson et al. 1995), and mammalian herbivores do not selectively avoid infected plants despite the alkaloid’s acute toxicity (Cook et al. 2009b; Harrison et al. 2021). The severe poisoning that swainsonine causes in livestock appears to be incidental rather than the product of selection on plant defense. If endophyte infection leaves no signature on growth, fecundity, or herbivore interactions, the relevant phenotypic consequences may lie in traits that have not yet been measured.

Two considerations motivate looking further. First, the same nine-year common garden that found no endophyte effect on survival or fecundity did detect more reproductive stems and more flowers per stem in E + *O. sericea*, and its authors identified pollination as specifically untested (Keith et al. 2025). Second, placing an association on the mutualism-commensalism-parasitism continuum requires evidence about the traits through which a host engages its community, not only about whole-plant fitness, and floral signaling in this system has never been measured. A well-supported null on those traits is therefore informative, because it constrains where the association sits rather than leaving the question open on grounds of an incomplete measurement.

One way in which *O. sericea* engages with its community is through emitted volatile organic compounds (VOCs). These compounds mediate interactions with pollinators, herbivores, and pathogens, and their production is highly plastic, shifting with tissue type, phenology, and abiotic conditions (Turlings et al. 1990; Raguso 2008; Campbell et al. 2019). Microbial partners reshape these emissions: mycorrhizal colonization, bacterial endophytes, and foliar fungal infections all alter the quantity and composition of host volatiles, with downstream consequences for pollinator attraction and indirect defense (Fontana et al. 2009; Pangesti et al. 2015). The closest precedent to the locoweed system comes from grass-endophyte associations, where alkaloid production is accompanied by shifts in emitted volatiles that modulate plant-insect interactions (Mwangi et al. 2024; Deng et al. 2024). Whether a comparable chemical signature exists in locoweeds is unknown; VOC profiles have not been characterized in *O. sericea* with respect to endophyte status, in either vegetative or floral tissue.

*O. sericea* then offers a tractable case: a long-lived legume with a vertically transmitted endophyte, naturally occurring infected and uninfected individuals, and a systemic alkaloid whose biosynthesis could plausibly intersect with volatile metabolism, as does in grasses-endophytes. Swainsonine is derived from lysine via pipecolic acid (Guan et al. 2021) and shares no immediate biosynthetic precursors with the isoprenoid (MVA/MEP) and lipoxygenase pathways that produce many plant volatiles (Dudareva et al. 2013), so any interaction is unlikely to be biosynthetic. Two indirect routes are nonetheless plausible. Alkaloid synthesis draws host nitrogen and carbon that would otherwise be available for other secondary metabolism, and fungal colonization can alter host defense signaling in ways that shift volatile emission independently of the alkaloid itself. Both routes are documented in grass-endophyte associations, where *Epichloë* infection changes emitted volatile profiles alongside alkaloid production and modifies plant-insect interactions (Mwangi et al. 2024; Deng et al. 2024). Here, we characterize the floral and foliar VOC emissions in *O. sericea* with and without *A. oxytropis* infection across greenhouse and common-garden plants. These data are paired with pollinator visitation, nectar rewards, auto-fertility tests, and tissue-level swainsonine localization. We examine whether endophyte presence shifts the chemical, reproductive, or attractive phenotype of the host, and whether any observable variation in these traits is better explained by plant-level differences unrelated to symbiotic status. Together, these comparisons test whether a relationship that leaves no mark on conventional fitness traits nonetheless reshapes the phenotypic axes through which the host engages its ecological community.

*O. sericea* ranges from the Canadian prairie provinces south through the Rocky Mountains and western Great Plains and occupying dry, well-drained foothill to montane grassland and sagebrush steppe (Esser 1993). Plants arise from a woody taproot and crown, producing odd-pinnate silvery leaves and leafless flowering scapes each spring (Cook et al. 2009b). Individuals are long-lived and slow to mature, with seedlings typically requiring two or more growing seasons before first flowering (Keith et al. 2025). The endophyte was originally described as *Undifilum oxytropis* (Pryor et al. 2009) but is now treated as *A. oxytropis* following the synonymizing of *Undifilum* under *Alternaria* (Woudenberg et al. 2013). Flowering occurs from early to late June and is primarily pollinated by bumble bees, particularly *Bombus flavifrons* (Kudo and Harder 2005).

## Methods

### Plant Material

Seedling recruits emerged naturally between 2021 and 2023 in a garden established at the Arthur H. Post Agronomy Farm (45°40’37.7"N 111°09’31.8"W) near Bozeman, MT. These recruits were progeny of 150 paired locoweed plants planted during a decade-long (2011–2020) common garden (Keith et al. 2025). Plants were grown from *O. sericea* seed field-collected from more than 100 individual plants at a single site in northeastern New Mexico (36°24’50.9"N, 103°39’57.8"W; Delaney et al. 2011), within the species’ native range.

One member of each pair retained its fungal endophyte (E+), while the other was rendered endophyte-free (E-). Because *A. oxytropis* resides in the parenchyma and aleurone layers of the seed coat and is transmitted to the seedling during germination (Oldrup et al. 2010; Ralphs et al. 2011), removing the seed coat eliminates the fungus. Seed coats and residual parenchymatous tissue were stripped with sterile tweezers, and the resulting embryos were surface sterilized in 1% bleach for 30 s and rinsed in sterile deionized water (Klypina et al. 2017). Mechanical removal was chosen over fungicide treatment because the two yield comparable results without exposing the embryo to fungicide (Grum et al. 2012), and seedlings emerged similarly regardless of seed coat manipulation (Keith et al. 2025). Seedlings were tagged at emergence to track individual plants, and swainsonine content was analyzed to determine endophyte presence in each. Across 2021–2023, a total of 1147 seedlings emerged and were monitored (E- = 671; E + = 476).

Greenhouse-grown *O. sericea* seedlings were established from surface-sterilized E + and E- seeds germinated on sterile, water-moistened filter paper at room temperature. Upon emergence of the first true leaf, seedlings were transplanted into Jiffy 7 peat pellet (Plantation Products, LLC, Norton, Massachusetts, USA) and thereafter potted into a uniform substrate consisting of Bozeman silt loam, Sunshine Mix no. 1 (Sun Gro Horticulture, Bellevue, Washington, USA), perlite, and washed fine sand (3:1:1:1 by volume). Plants were maintained under a 16 h photoperiod (natural light supplemented with mercury-vapor lamps at ~ 165 µmol m^− 2^ s^− 1^) in a greenhouse held at 25 ± 4 °C and watered and fertilized biweekly with Jack’s Classic 20-20-20 (N-P-K) All Purpose fertilizer (J. R. Peters, Inc, Allentown, Pennsylvania, USA) solution at 100 ppm. Seedlings were started in July, and endophyte status was confirmed for every plant by both PCR and swainsonine assays (USDA-ARS, Logan, UT) prior to volatile collections as detailed in Keith et al. (2025).

Pairing in this study was spatial and phenotypic rather than genetic. Founder plants established in 2011 were paired at the seed-lot level, with E- individuals generated by removing the seed coat from seed of the same accession, so Gen 0 pair members shared close genetic background. From Gen 1 onward, however, E + and E- lines were propagated separately from seed collected on plants of the corresponding status, and the plants sampled here were open-pollinated recruits of those lines. Paternity was uncontrolled, and we did not genotype individuals. Field pairs were therefore formed at the time of sampling by selecting two similarly sized plants of opposing endophyte status within a 5-m radius, and greenhouse pairs by matching adjacent plants of similar size and developmental stage.

### Volatile Organic Compound Collection

#### Field Collection

We used a pull sampling technique similar to that described in Perez (2009) and Malone et al. (2022). VOCs were collected from the headspace of chambers constructed from plastic oven bags (Reynolds Consumer Products, Louisville, KY). Bags were washed and baked at 200 °C in an autoclave oven prior to volatile collection to remove any residual volatiles from manufacturing. VOCs were collected using a custom-built, portable volatile collection system, powered by a 12 V / 5 A Duracell^®^ Ultra sealed battery. The system included a 12 V diaphragm vacuum pump (Model 5002 − 0408 DC-PM, 3000 rpm, Rietschle Thomas) controlled via a basic on/off light switch, which provided the vacuum needed to draw air through the system. Airflow was regulated using a pair of flowmeters (Series MR 3000, Model 3A13, Key Instruments) connected via Tygon^®^ tubing. Each sampling line consisted of a 1.5 m Tygon^®^ tube (0.95 cm OD x 0.64 cm ID) leading from the flowmeters to a volatile collection trap (6.35 mm OD x 76 mm length, glass; Analytical Research Systems, Inc.) filled with 30 mg of Super-Q adsorbent (Alltech Associates, Deerfield, IL). VOCs were adsorbed onto Super-Q during sampling and eluted immediately after each collection period. The volatile collection apparatus was enclosed in a plastic bucket (25 × 25 × 20 cm) containing a smaller metal housing (15 × 15 × 7 cm) that held the flowmeters and associated tubing. Tygon^®^ tubing extended from the metal housing to individual collection traps positioned in the field.

For each sampling event, two similar plants of opposing endophyte status (as previously determined by lab sampling) were selected within a 5-m radius. This paired design controls for microsite heterogeneity by placing E + and E- individuals in immediate proximity, providing a test of symbiotic effects on plant chemistry. Collections were made from three stem types: green stems, flowering stems, senescing flowered stems. For each collection type, five stems were randomly selected. The oven bag was placed over the selected stems to create a collection chamber. A small hole was cut at the top of each bag to allow airflow, and the base of the bag was secured around the stems using a twist tie. The collection apparatus bucket was positioned between the paired plants, and the Tygon^®^ tubing was connected from the flowmeters to the volatile traps. The traps were inserted vertically into the oven bags, positioned among the stems to capture emitted volatiles. Leaf volatiles were collected in May, and flower volatiles were collected in June. Following each 2-hour collection period, traps were removed, labeled, wrapped in aluminum foil, and transported to the laboratory for elution. After trap removal, the stems enclosed in each bag were clipped at the base of the oven bag, harvested, and returned to the lab for fresh weight measurements.

#### Greenhouse VOC Collection

VOC collections were carried out using a protocol consistent with that described by Weaver et al. (2009). Headspace volatiles were collected over a 6-hour period from 09:00 to 15:00 in April. For each trial, 12 individual plants were sampled simultaneously in a paired design: each pair consisted of two neighboring individuals matched on endophyte status and approximate plant size, one E + and one E-, positioned adjacent to one another in the greenhouse to standardize soil, moisture, and light conditions between pairs. This design allowed differences in volatile profiles to be attributed more confidently to endophyte status rather than microsite variation.

Volatiles were captured using 80-cm long x 4-cm diameter glass volatile collection chambers, each fitted with a threaded 10-cm diameter top port. An 8-port glass manifold adapter was secured to the top of each chamber. Two of the manifold’s ports held glass traps (6.35 mm x 76 mm; Sigma Scientific, Gainesville, Florida), each packed with 30 mg of HayeSep-Q adsorbent (Hayes Separation, Inc., Bandera, Texas), while the remaining ports were sealed to prevent outside air entry and pressure loss. One trap was used to collect background volatiles for 10 min during system equilibration and was discarded, while the second collected plant-emitted volatiles during the subsequent 6-hour sampling period. Clean, humidified, and charcoal-filtered air was delivered to each chamber via Teflon tubing at a flow rate of 1 L/min using a diaphragm air pump. At the outlet, a vacuum pump was connected to the traps via Teflon tubing to maintain airflow and pressure. The base of each plant stem was sealed using a Teflon guillotine clamp, with a small piece of cotton wrapped around the stem to ensure an airtight seal and minimize ambient or soil-derived contamination. At the conclusion of each 6-h collection, the enclosed aboveground tissue was excised at the guillotine clamp and weighed fresh to standardize emission rates.

#### VOC Elution and Analysis

Following field and greenhouse collections, VOCs were eluted from the traps with 200 µl aliquot of dichloromethane. Elution was performed slowly, with nitrogen gas used to gently push the solvent through the trap and into a glass insert inside a 1.5-ml crimp-sealed vial. An internal standard of 10 µl nonyl acetate was added to each sample to facilitate compound quantification.

Samples were analyzed using gas chromatography-mass spectrometry (GC-MS). Chromatographic separation was performed on an HP-5MS column (30 m x 0.25 mm, 0.25 μm film; J&W Scientific, Folsom, CA) installed in an Agilent 6890 GC coupled to an Agilent 5973 mass spectrometer (Agilent Technologies, Santa Clara, CA). Sample injection was automated using pulsed splitless mode at an inlet temperature of 250 °C and an initial pressure of 82.7 kPa. The oven temperature was programmed to hold at 50 °C for 4 min, increase by 5 °C per minute to 160 °C, then ramp at 25 °C per minute to a final temperature of 280 °C. The transfer line temperature was set to 300 °C. Helium served as the carrier gas with a constant flow rate of 1.2 ml/min. The mass spectrometer was operated in SCAN mode from 50 to 300 m/z. Compound identification was achieved through comparison of retention times and mass spectra with entries in the NIST spectral library. Identities of the seven compounds were further confirmed by comparison of retention times and mass spectra with those of authentic reference standards (Sigma-Aldrich, St. Louis, MO) analyzed under identical instrumental conditions. Data acquisition and peak integration were conducted using MSD ChemStation software (Agilent Technologies).

Because plastic oven bags were used for field headspace chambers, we implemented explicit artifact screening and cross‑validation steps. Prior to analysis, all compounds present in bag‑only blank collections were flagged and compared against sample peaks by retention time and full‑scan mass spectra. Any compound showing even partial overlap with bag‑derived spectra or plasticizer signatures was conservatively excluded from biological analyses. To verify that biologically relevant compounds retained for analysis were not artefactual, we cross‑validated the field detections against greenhouse glass‑chamber collections: Each of the seven core compounds retained in field analyses was independently confirmed in greenhouse headspace samples collected with inert glass chambers and charcoal‑filtered inlet air, confirming their biological origin. For low‑abundance or chemically labile peaks, inclusion required three independent criteria: (i) NIST spectral match > 85%, (ii) consistent retention index within ± 2% of greenhouse standard runs, and (iii) presence in at least two independent biological replicates. Together, these steps provide robust assurance that all compounds reported represent authentic plant emissions, not sampling artifacts.

### Pollinator Visits and Nectar Analysis

During peak bloom in June 2023 and 2024, pollinator activity was recorded on clear mornings between 09:00 and 11:00. Each observation block lasted 10 min, and floral visits, defined as any contact between an insect and reproductive structures, were tallied, yielding a total of 17.5 h in 2023 and 16.2 h in 2024. Nectar was sampled between 08:00 and 10:00 using calibrated microcapillary tubes; volumes (in µL per flower), and sucrose concentration (% w/v) were measured immediately with % sucrose determined with a handheld refractometer (Bellingham & Stanley, Xylem Inc., Washington, DC, USA). Volatile collections were conducted in 2022 and 2023; pollinator observations extended through 2024 to increase temporal replication of visitation data and test whether the modest 2023 endophyte-visit association was repeatable across years.

### Pollinator Exclusion to Test Plant Auto-Fertility

To assess autonomous self-pollination (i.e. self-fertilization without insects or wind), we selected 20 pairs of E + and E- plants in June 2023 and enclosed their pre‑flowering scapes in bridal‑veil bags for one month to exclude insect visitors. To compare autonomous self-pollination, and to test whether endophyte status affected seed set under natural pollinator access, another 20 pairs of E + and E- plants were randomly selected in August 2023. Scapes were bagged only after flowering, ensuring seed pods were retained until dehiscence. Following pod maturation, all seeds were harvested and total seed set per scape was counted to determine whether pollinator visitation was required for fertilization and whether E + and E- differ in seed set.

### Swainsonine Content

Swainsonine concentrations in leaves and dissected floral organs were quantified by extracting 25–50 mg dried, ground tissue in 2% acetic acid for 18 h, followed by LC-MS analysis (Gardner et al. 2001) at the USDA-ARS facility in Logan, UT. Quantitation was achieved using an external calibration curve with detection limits of 0.001% dry mass. During peak flowering, eight E+ plants were randomly selected, and 20 scapes from each were dissected into calyces, petals, pistils, and pollen. Nectar was collected as a pooled sample from approximately 40 E+ plants (40 µL total) and analyzed for swainsonine content.

### Data Analysis

Volatile compound concentrations were expressed per unit plant biomass per hour (ng g⁻¹ h⁻¹). Immediately following each collection period, enclosed tissue was clipped at the base of the chamber and weighed fresh to obtain the biomass denominator. Raw (untransformed) values were used for concentration estimates, while compositional data were transformed using a centered log-ratio (CLR; R package *compositions* v2.0-9; R v4.5.1, R Core Team) transformation (Brückner and Heethoff 2017). Known background compounds and analytical artifacts identified in bag blanks were removed prior to compositional analyses. For borderline low-intensity peaks, we required co-occurrence in greenhouse samples and concordant retention index and mass spectrum before inclusion in statistical analyses.

Multivariate differences in volatile profiles due to endophyte status, tissue type, and pair identity were assessed using permutational multivariate analysis of variance (PERMANOVA) based on Euclidean distances of CLR-transformed data with 999 permutations using the adonis2 package (vegan v2.7-2; Oksanen et al. 2026) in R. Euclidean distance was chosen because the CLR transformation converts compositional data to real space, making Euclidean distances appropriate for assessing multivariate differences. Models were fit using marginal sums of squares (Type III), with permutations constrained within year (strata = year) to account for temporal structure; year was therefore not included as a model term. Fixed effects included endophyte status (E+/E-) and, where applicable, collection type (leaf versus flower), with plant pair included as a fixed term in the model to account for the paired E+/E- design. As a check, we refit the model as a distance-based redundancy analysis (dbRDA; capscale, vegan) with pair identity specified as a conditioning variable rather than a model term. Results were equivalent (identical F-statistics; p-values differing by less than 0.03), so inference for endophyte status was insensitive to how pair identity was handled.

Homogeneity of multivariate dispersion among groups was verified using betadisper (vegan v2.7-2) to confirm that PERMANOVA results reflected centroid differences rather than dispersion effects, and to test whether among-individual variation differed in VOC composition differed between endophyte groups. Dispersion was tested separately for year, endophyte status, and tissue type; because betadisper operates independently of the PERMANOVA permutation scheme, year could be included as a grouping factor in dispersion tests even though it was excluded as a model term from the PERMANOVA.

PCA was used to visualize multivariate structure in VOC composition. PCA was computed on CLR-transformed profiles using prcomp (R Core Team 2026), with compounds centered and scaled to unit variance so that no single compound dominated the ordination by virtue of its emission magnitude. Separate ordinations were computed for the greenhouse and field datasets. For the field ordination, all three tissue types (flowers, living leaves, and senesced leaves) were included so that the full compositional spread across tissues could be displayed on common axes.

Because PERMANOVA tests for overall compositional differences without indicating which compounds contribute to the observed dissimilarity, we used similarity percentage (SIMPER) analysis to examine whether any individual compound showed a consistent directional difference between endophyte groups that a multivariate test might obscure. SIMPER partitions dissimilarity regardless of whether that dissimilarity is statistically supported, so we treated its output as a descriptive check on the multivariate result rather than as a test of endophyte effects. Analyses were conducted on untransformed data using Bray-Curtis dissimilarities (vegan v2.7-2). CLR-transformed values were not used for SIMPER because they can include negative components, violating the assumptions of the Bray-Curtis metric.

To test whether endophyte status affected total volatile emission rates, we fit linear mixed-effects models (LMM) with log-transformed total VOC concentration (ng g⁻¹ h⁻¹) as the response variable using the R package lme4 (Bates et al. 2015), with p-values derived via Satterthwaite’s method (lmerTest; Kuznetsova et al. 2017). Fixed effects included endophyte status and, where applicable, collection type (leaf vs. flower); year was included as a fixed effect in the combined model. Plant pair was included as a random intercept to account for the paired E+/E- design. Models were fit separately for greenhouse and field datasets, and year-stratified models were run to assess consistency across sampling periods.

Model assumptions were checked for all analyses. For the linear mixed-effects models, residuals were inspected for normality and homoscedasticity using quantile-quantile and residual-versus-fitted plots, and the log transformation of total VOC concentration was applied to satisfy these assumptions. For the PERMANOVA and dbRDA analyses, the assumption of homogeneous multivariate dispersion was assessed with betadisper as described above. No violations were detected that would alter the interpretation of our results.

To characterize the sensitivity of our paired PERMANOVA design, we conducted a simulation-based power analysis in R. We generated 200 simulated datasets per effect size by adding progressively larger numeric shifts to the VOC profiles of endophyte-positive samples, scaled to the empirical within-pair standard deviation of CLR-transformed compounds. Each simulated dataset was analyzed using the same PERMANOVA model applied to the observed data. The proportion of significant results across simulations at each effect size reflects the probability that our design would detect a true compositional difference of that magnitude.

We tested the effect of endophyte status on pollinator behavior and nectar characteristics – specifically, the number of flowers probed per plant, nectar sucrose concentration (%), and nectar volume – using separate LMMs accounting for year, seasonality, and time of day.

## Results

### Volatile Organic Compound Profiles

#### Shared VOCs Across Environments

Seven volatiles, (*Z*)-3-hexenyl acetate, 6-methyl-5-hepten-2-one, decanal, methyl salicylate, nonanal, octanal, and β-ocimene, were detected under both greenhouse and field conditions. In all cases, relative abundances of these compounds did not differ by endophyte status (PERMANOVA: greenhouse *p* = 0.798, field *p* = 0.171; LMMs: all *p* > 0.10), underscoring that factors associated with plant pair identity, tissue type, and year overshadow any detectable influence of *A. oxytropis* on VOC emissions.

#### Greenhouse-Grown Plants

**Fig. 1:**
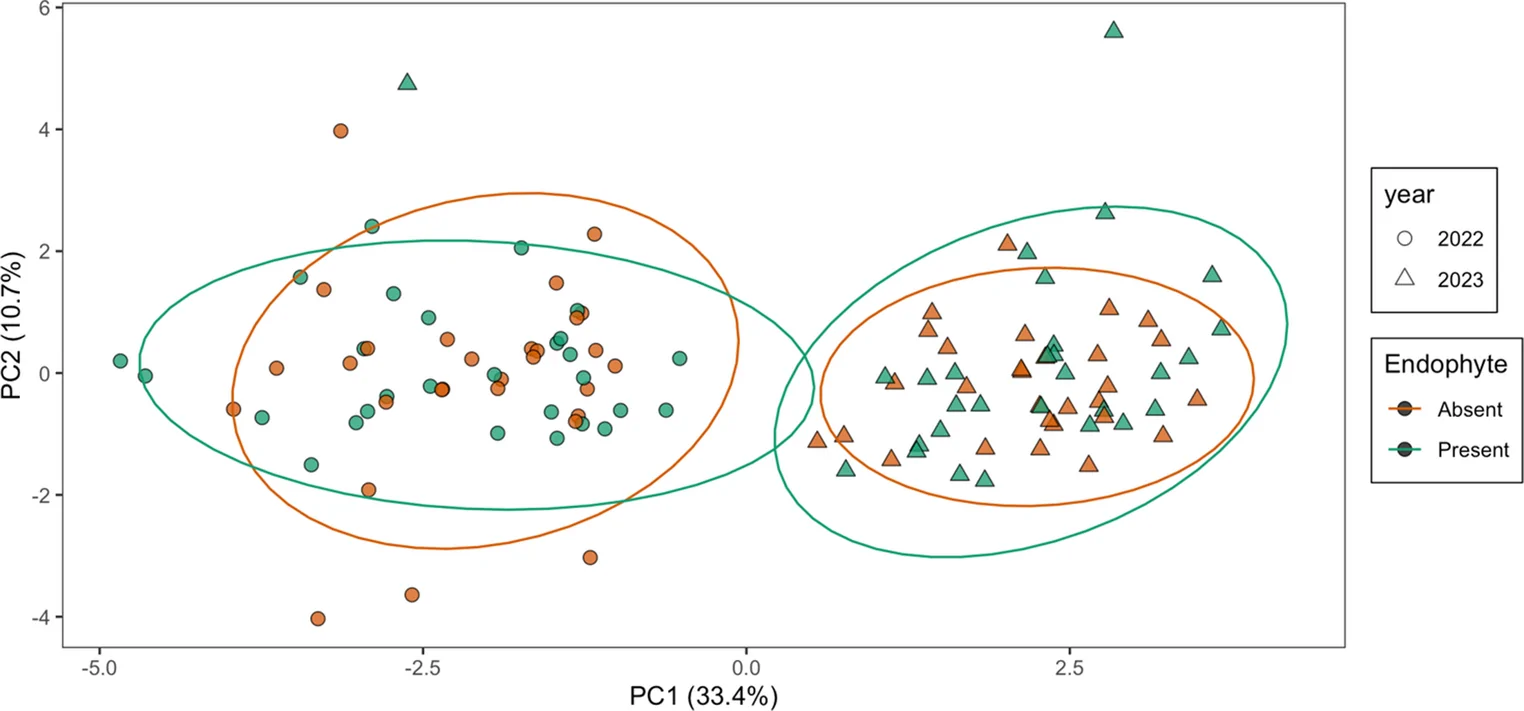
Principal component analysis of foliar volatile organic compound (VOC) composition in *Oxytropis sericea* greenhouse plants (Spring 2022, *n* = 57; Spring 2023, *n* = 58). Each point represents an individual plant; point shape indicates collection year and ellipses denote 95% confidence regions for each year. Point color indicates *Alternaria oxytropis* endophyte status (orange = absent, green = present). VOC compositions were CLR-transformed prior to analysis. PC1 and PC2 explain 33.4% and 10.7% of total compositional variance, respectively

GC‑MS identified 22 VOCs across seven chemical classes (Supp. Table 1). Plant pair identity (PERMANOVA: R² = 0.726, F = 2.453, *p* = 0.003) influenced VOC composition, while endophyte status had a negligible effect (R² = 0.003, F = 0.636, *p* = 0.798). Among-individual variation in VOC composition by year (*p* = 0.048) but not by endophyte status (*p* = 0.814); PERMANOVA ellipses differences for pair should therefore be interpreted with this dispersion asymmetry in mind. PCA captured 33.4% (PC1) and 10.7% (PC2) of the variance (Fig. 1). PC1 was driven by dodecanal, methyl salicylate, and octanal, while PC2 reflected variation in nonanal, decanal, and 6-methyl-5-hepten-2-one (PCA loadings, prcomp).

#### Field-Grown Plants

**Fig. 2:**
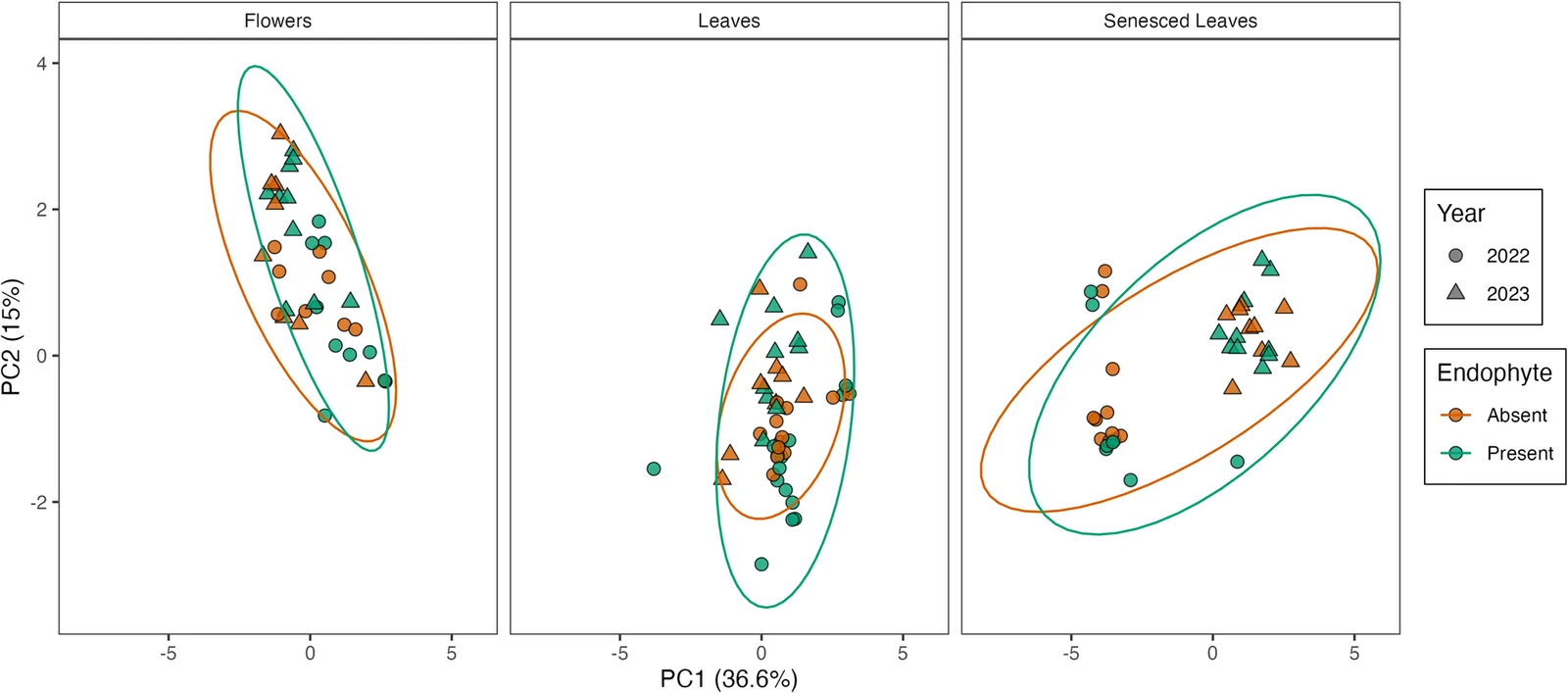
Principal component analysis of VOC composition in field-collected *Oxytropis sericea* tissues from a common garden at Post Farm (Bozeman, MT) in 2022 and 2023, faceted by tissue type (Flowers: *n* = 36; Leaves: *n* = 48; Senesced Leaves: *n* = 37). Each point represents an individual sample; point shape indicates collection year (circles = 2022, triangles = 2023). Ellipses denote 95% confidence regions for each endophyte status group. Point color indicates *Alternaria oxytropis* endophyte status (orange = absent, green = present). VOC compositions were CLR-transformed prior to analysis

In field samples, 42 VOCs were initially detected, with 10 compounds retained for analysis based on consistency and biological relevance (Supp. Table 2). Critically, the seven core compounds were cross-validated against the greenhouse collections using inert glass chambers, confirming their biological origin rather than being contamination artifacts. Plant pair identity strongly influenced VOC composition (PERMANOVA: R² = 0.649, F = 2.226, *p* = 0.001), while collection type (R² = 0.004, F = 0.676, *p* = 0.594) and endophyte status (R² = 0.009, F = 1.534, *p* = 0.171) did not. Among-individual variation in VOC composition did not differ by year (betadisper: *p* = 0.089), tissue type (*p* = 0.164), or endophyte status (*p* = 0.756), indicating that PERMANOVA results reflect centroid differences rather than dispersion effects. PCA of the CLR-transformed VOCs captured 25.9% and 19.6% of the total variance on PC1 and PC2 (Fig. 2), respectively.

For field-collected senesced leaves (*n* = 37; 2022: E- = 10, E + = 8; 2023: E- = 9, E + = 10), endophyte status had no significant effect on VOC composition after accounting for pair identity (PERMANOVA: R² = 0.004, F = 0.428, *p* = 0.85), and betadisper showed no significant heterogeneity in dispersion by year (*p* = 0.181) or endophyte status (*p* = 0.469).

**Fig. 3:**
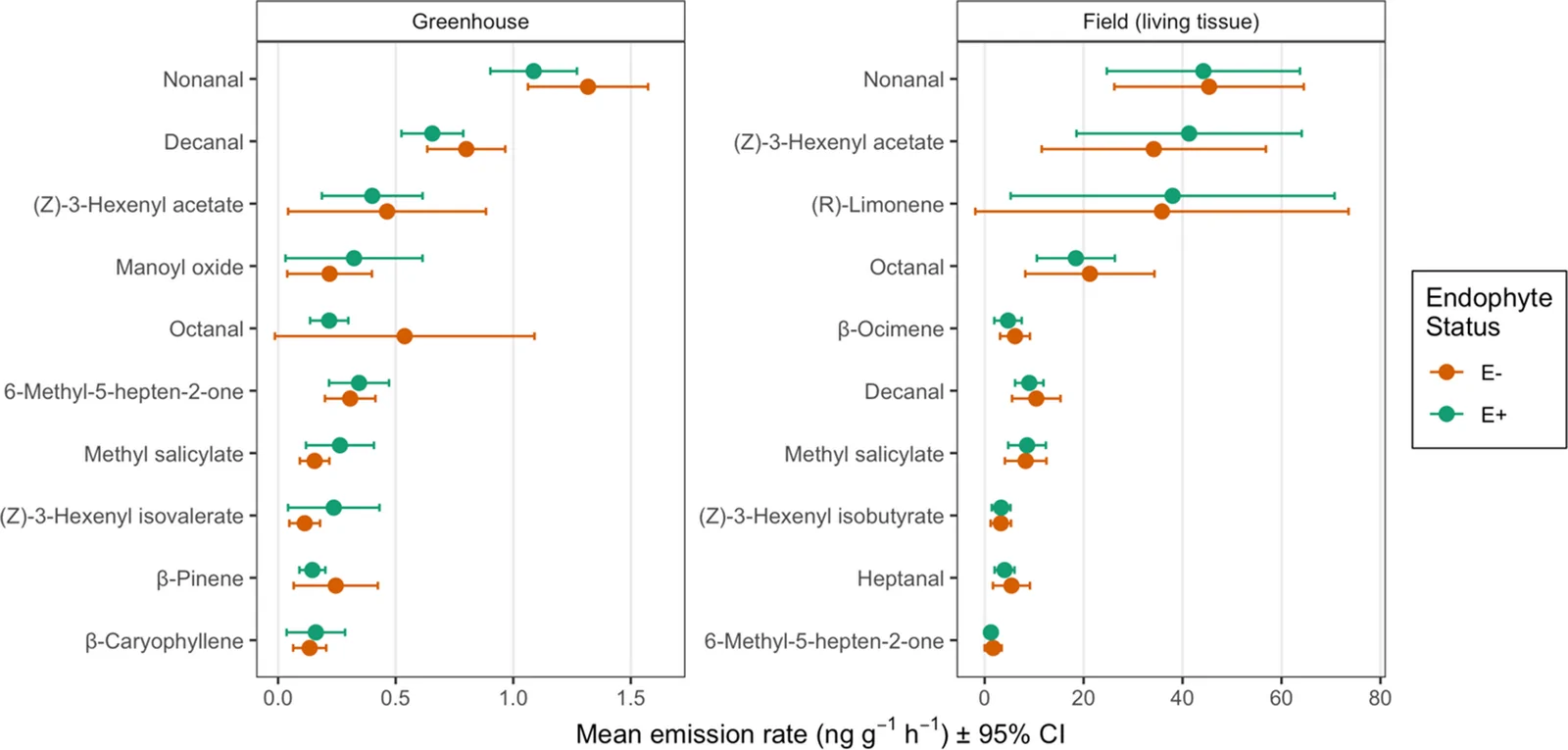
Mean volatile emission rates (ng g⁻¹ h⁻¹, ± 95% CI) for the compounds contributing most to Bray-Curtis dissimilarity between endophyte-absent (E-) and endophyte-present (E+) *Oxytropis sericea* plants, in greenhouse (left) and field living tissue (right) datasets. Compounds are ordered top to bottom by SIMPER contribution rank. Means and confidence intervals are calculated from raw emission values. SIMPER partitions dissimilarity between groups regardless of whether that dissimilarity is statistically supported, so contributions reflect compound abundance rather than endophyte-associated differences. No compound differed significantly by endophyte status

SIMPER was used to ask whether any single compound showed a consistent directional difference between endophyte groups despite the non-significant multivariate result. It did not. The compounds contributing most to Bray-Curtis dissimilarity were nonanal (average contribution 0.109) and decanal (0.073) in the greenhouse and nonanal (0.175), (Z)-3-hexenyl acetate (0.171), and (R)-limonene (0.131) in field living tissue (Fig. 3). These are the most abundant compounds in each dataset, and their high contributions reflect emission magnitude rather than any endophyte-associated shift, since SIMPER partitions dissimilarity irrespective of whether that dissimilarity is statistically supported. Across both datasets, compounds were roughly equally likely to be more abundant in E + or E- plants, with no directional signal by endophyte status (Fig. 3). Linear mixed models fit to the two highest-ranked compounds in each dataset confirmed this: endophyte status had no significant effect on log-transformed abundance in any case (all *p* > 0.10), with the strongest trend being a non-significant reduction of nonanal in greenhouse E+ plants (estimate = -0.18, t = -1.58, *p* = 0.12). Year was a significant predictor in all models, consistent with the strong inter-annual variation in the multivariate analyses. Total VOC emission rates likewise did not differ between E- and E+ plants in either the greenhouse (combined: β = −0.091, SE = 0.130, df = 58.0, t = − 0.696, *p* = 0.489) or field (combined: β = 0.175, SE = 0.243, df = 44.6, t = 0.720, *p* = 0.475), and this pattern held within each year separately (all *p* > 0.38).

#### Power Analysis

Our simulation-based power analysis indicated that the paired PERMANOVA design reached 80% power to detect shifts as small as 0.16 times the empirical within-pair standard deviation in CLR-transformed VOC profiles. To place this in context, a shift of that magnitude is approximately 15% of the typical compositional distance separating two individual plants in our dataset, so the design was sensitive to endophyte effects several times smaller than the among-individual variation that dominates the data. The lack of a significant effect of endophyte status is therefore unlikely to be explained by insufficient statistical power, and any undetected effect, if present, is likely to be small.

### Floral Swainsonine Content

**Fig. 4:**
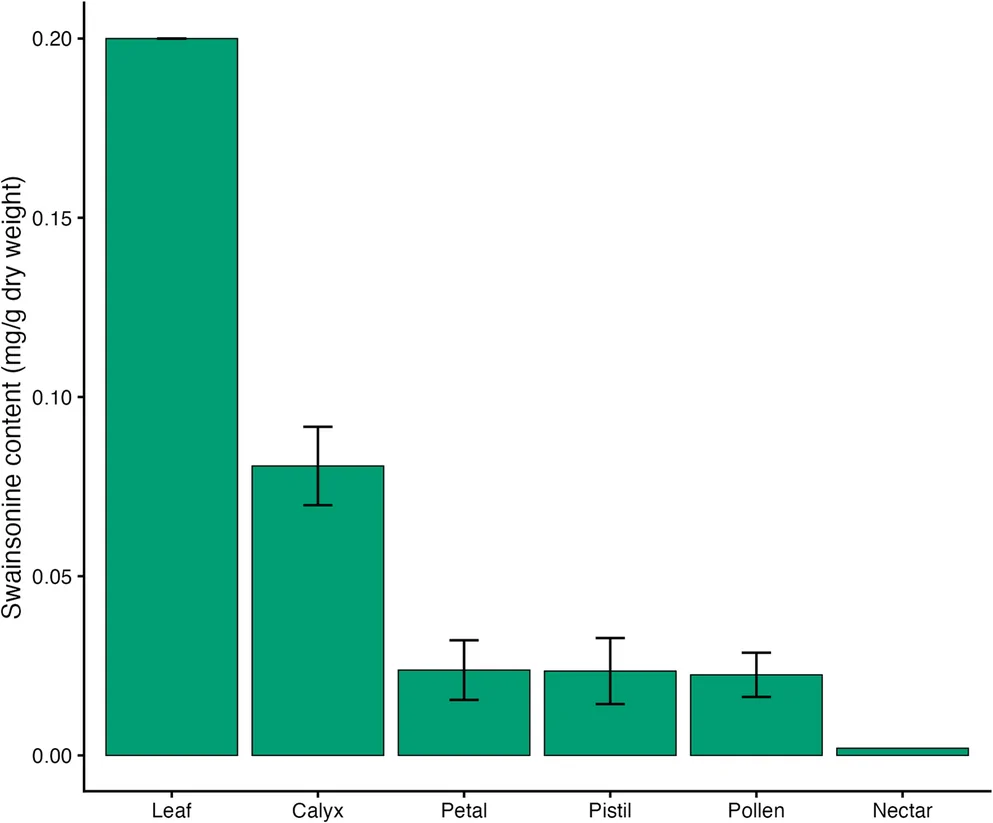
Swainsonine content (mg g⁻¹ dry mass) in leaves and dissected floral organs of *Alternaria oxytropis*-infected (E+) *Oxytropis sericea* plants. During peak flowering, eight E+ plants were randomly selected and 20 scapes per plant were dissected into calyces, petals, pistils, and pollen; nectar was collected as a single pooled sample from approximately 40 E+ plants. Error bars represent ± 1 SE; no error bar is shown for nectar, which was analyzed as a single pooled sample. Swainsonine was undetectable in E- plants. Leaf concentrations were approximately an order of magnitude higher than any floral tissue; nectar contained near-trace concentrations just above the LC-MS quantification limit of 0.001% dry mass

Swainsonine was detected in all dissected floral organs of E+ plants at concentrations approximately 10-fold lower than in leaves (0.200 ± 0.008 mg g⁻¹ ; Fig. 4). Concentrations were highest in calyces (0.081 ± 0.010 mg g⁻¹ dry mass), with lower but equivalent levels in petals (0.025 ± 0.008 mg g⁻¹ ), pistils (0.023 ± 0.004 mg g⁻¹ ), and pollen (0.022 ± 0.006 mg g⁻¹ ). The single, pooled nectar sample from ca. 40 flowers contained near trace swainsonine (< 0.002 mg g⁻¹, just above the LC-MS quantification limit of 0.001 mg g⁻¹ ).

### Pollinator Observations

**Fig. 5:**
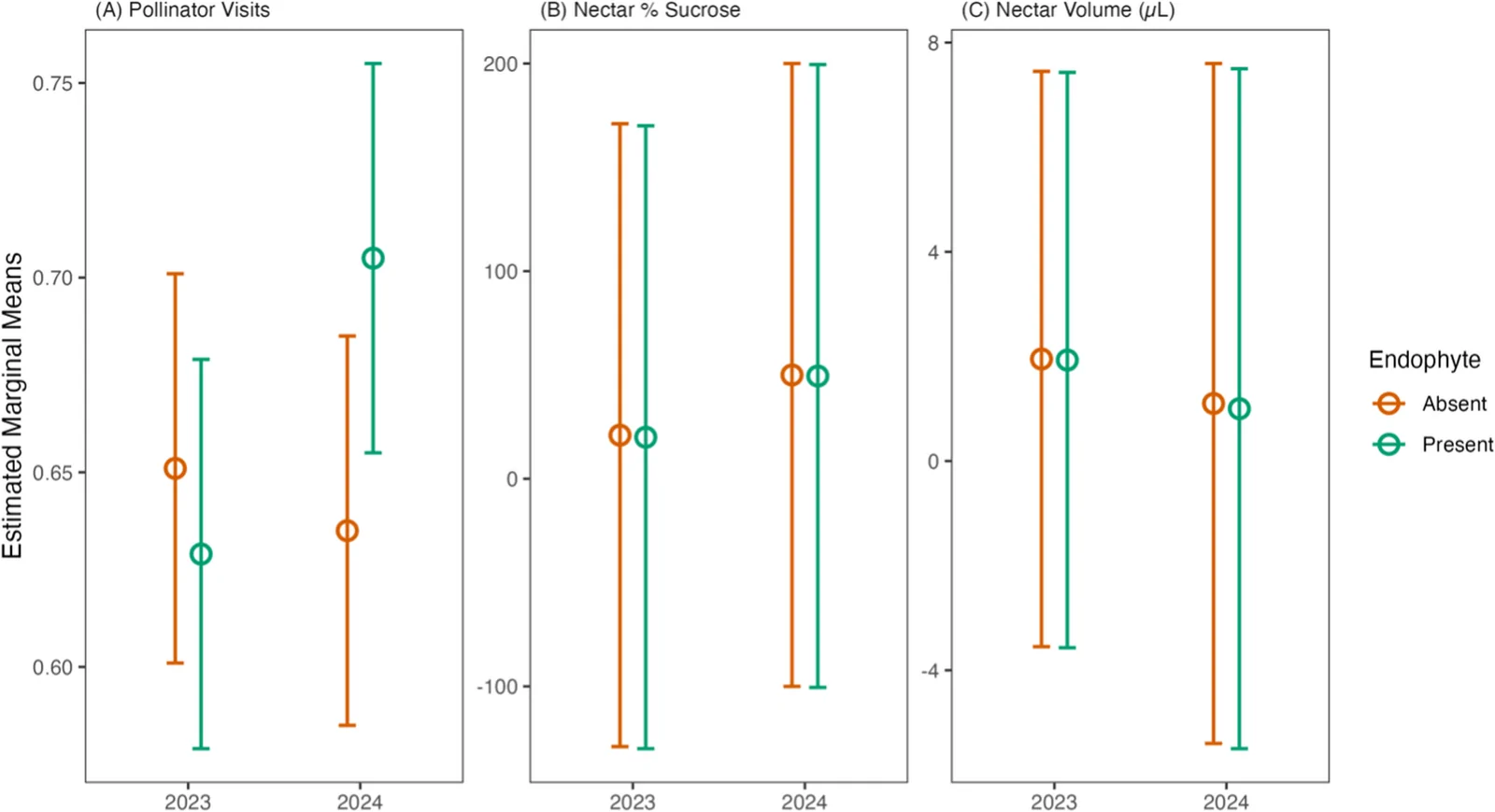
Estimated marginal means (± 95% CI) from linear mixed-effects models for (**A**) pollinator visits per plant, (**B**) nectar sucrose concentration (% w/v), and (**C**) nectar volume (µL) in endophyte-infected (E+, green) and endophyte-free (E−, black) *Oxytropis sericea* plants across observation years (2023–2024)

We recorded 9,627 (9.17 visits/min) bee visits in 2023 and 9,206 (9.47 visits/min) in 2024. Native bees, *Bombus huntii*, *B. nevadensis*, and several male *Megachile spp.* dominated the assemblage based on visual identification (personal communication, Casey Delphia, 2023). In 2023, mean visits per plant did not differ between E+ (*n* = 4,812) and E- (*n* = 4,815) plants (F_1,30_ = 0.12, *p* = 0.73; Fig. 4). In 2024, E+ flowers received a modest increase in visits compared to E- plants (mean ± SE: 5.12 ± 0.21 vs. 4.08 ± 0.18 visits per plant; F_1,30_ = 6.48, *p* = 0.016, Fig. 5), with no corresponding differences in VOC or nectar traits.

### Nectar Volume & Sucrose Concentration

Nectar averaged 2.03 ± 0.09 µL per flower for E+ plants and 2.07 ± 0.08 µL for E- plants, with no significant effect of endophyte status after accounting for year, time of day, and plant pair (F_1,58_ = 0.07, *p* = 0.79; Fig. 5). Similarly, sucrose concentration did not differ (E+: 32.6 ± 1.2%; E-: 34.9 ± 1.1%; F_1,58_ = 1.34, *p* = 0.25, Fig. 5). Across treatments, nectar volume and sucrose concentration were negatively correlated (*r* = -0.47, *p* < 0.001).

### Pollinator Exclusion and Outcrossing

Bagged flowers on both E + and E- plants failed to set seed, whereas all unbagged controls produced pods, confirming that insects are required for pollination in *O. sericea*. Mean seed set per scape did not differ by endophyte status in open-pollinated flowers (E+: 28.4 ± 2.1 seeds; E-: 29.1 ± 2.3 seeds; F_1,38_ = 0.05, *p* = 0.82).

## Discussion

Despite swainsonine’s well-documented toxicity to livestock and the nitrogen the host must supply to sustain fungal alkaloid biosynthesis (Valdez Barillas et al. 2007; Guan et al. 2021), we detected no measurable influence of *Alternaria oxytropis* on any plant phenotype we examined. VOC composition and among-individual VOC variation were indistinguishable between endophyte-positive and endophyte-negative plants in both greenhouse and field settings, and floral chemistry, nectar rewards, pollinator visitation, and seed set were likewise unaffected. The dominant axes of variation in our data were instead inter-annual and among-individual: VOC profiles differed markedly between collection years, and a single year showed modestly elevated pollinator visitation to E+ plants with no accompanying shift in floral chemistry or nectar reward. Visitation was tallied per plant rather than per flower, and E + *O. sericea* in this garden system have been reported to produce more reproductive stems and more flowers per stem than their E- pairs (Keith et al. 2025). A larger floral display, rather than any difference in scent or reward, is therefore the more parsimonious explanation for the 2024 difference, and it locates the effect in inflorescence architecture rather than in chemical signaling. *O. sericea* requires insect visitors for pollination, yet open-pollinated seed set per scape was identical between symbiotic states. Together these findings are consistent with the *Oxytropis–Alternaria* association functioning as a commensalism. Because pair members were matched on microsite and size rather than genotype, host genetic variation among individuals cannot be separated from other sources of among-plant variation in our design and may contribute to the large pair effect we report.

The persistence of vertical transmission despite apparent host neutrality presents an evolutionary puzzle. One reading is that the endophyte imposes a real metabolic cost that is offset by a benefit we did not measure, yielding a net-neutral interaction. The available evidence, however, points towards a simpler explanation, that the cost itself is small. Swainsonine biosynthesis requires nitrogen, but nitrogen supplementation that raised *O. sericea* shoot biomass by 30 to 40% did not raise leaf swainsonine concentration, and total plant swainsonine increased less in *O. sericea* than in any other locoweed taxon tested (Delaney et al. 2011). Nor did elevated CO_2_ alter swainsonine production in two *Astragalus* congeners, *A. lentiginosus* and *A. mollissimus* (Cook et al. 2017), and water deficit produced no detectable swainsonine-growth trade-off (Vallotton et al. 2012). Relative endophyte biomass in aboveground *O. sericea* tissue is on the order of 0.5 to 4% (Cook et al. 2009a), comparable to *Epichloë* in perennial ryegrass. In a nine-year common garden, E + *O. sericea* plants were slightly larger in crown and stem mass than their E- pairs (Keith et al. 2025). The clearest evidence of a cost in this group comes from a congener, where *A. lentiginosus* grown with the endophyte had reduced plant volume and lower leaf nitrogen (Harrison et al. 2021), suggesting the balance of the association may differ among host species. We emphasize that no study has directly quantified the metabolic cost of hosting *A. oxytropis* in *O. sericea*. The evidence above is indirect, drawn from alkaloid responses to resource supplementation and from whole-plant fitness comparisons. Our inference that the cost is small therefore rests on the absence of any detected trade-off across several resource axes rather than on direct measurement.

Mathis and Bronstein (2020) distinguish two forms of commensalism that our data cannot yet separate. In no-effects commensalism, the unaffected partner experiences neither costs nor benefits, whereas in balanced-costs-and-benefits commensalism real costs and benefits cancel to a net effect of zero. The evidence reviewed above favors the no-effects form, since no cost has been detected across nitrogen, water, and CO_2_ manipulations, but a small, compensated cost cannot be excluded. Balanced-costs-and-benefits commensalisms require a precise cancellation that modest ecological variation can tip toward mutualism or antagonism, whereas no-effects commensalisms should be more evolutionarily stable and less context-dependent (Mathis and Bronstein 2020). If the *Oxytropis-Alternaria* association is a no-effects commensalism, its neutrality should hold across a broad range of conditions; if the costs and benefits merely balance, we would expect the outcome to shift with resource availability or herbivore pressure. Separating these requires direct measurement of host-to-fungus resource flux, for example isotopic labeling paired with tissue-specific transcriptomics, rather than inference from whole-plant fitness traits.

Endophyte benefits, if they exist, may be context-dependent rather than constitutive, but evidence for stress-mediated benefits in locoweeds remains limited: drought conditions altered antioxidant defenses in *Astragalus mollissimus* but not in *O. sericea* (Klypina et al. 2017), and a decade of variable environmental conditions detected no endophyte effect on host fecundity (Keith et al. 2025). The tissue localization of swainsonine – concentrated in calyces (0.081% dry mass) while virtually absent from nectar (< 0.002%) – likely reflects the physical distribution of fungal hyphae within reproductive tissues rather than an adaptive defensive strategy (Noor et al. 2018), contrasting sharply with alkaloid systems in *Nicotiana* and *Lupinus* where tissue partitioning serves clear defensive functions (Lee et al. 2007; Kessler et al. 2012; Adler et al. 2012; Otterbach et al. 2019).

Although swainsonine concentrations in individual seeds fall below reliable detection limits (Gardner et al. 2001), vertical transmission efficiencies nearing 100% (Ralphs et al. 2011) indicate that seeds carry both the fungus and its alkaloids; a decade of observation at the same common garden recorded negligible herbivory on seeds or foliage (Keith et al. 2025), offering no evidence that calyx-localized swainsonine functions in seed or bud defense. That the fungus gains shelter and vertical transmission while the host experiences no detectable fitness return places this association in a category that is common in description but rarely established with evidence. Commensalism is frequently invoked as a default when effects are not detected, and Mathis and Bronstein (2020) argue that most reported commensalisms are better described as interactions whose effects have not been measured with sufficient resolution, rather than interactions demonstrated to be neutral. Zapalski (2011) has argued on this basis that commensalism cannot be demonstrated at all, only inferred from failure to detect an effect, and Mathis and Bronstein (2020) concur that neutral effects must be measured with the same rigor applied to positive and negative ones. Our simulation-based power analysis indicates that compositional shifts well below the among-individual variation we observed would have been detected. Neutrality on the traits we measured is therefore a result rather than an absence of one. Whether that neutrality holds across environments is a separate question, since the outcome of many symbioses shifts with context (Bronstein 1994; Chamberlain et al. 2014), and fungal endophyte associations in particular span a continuum from mutualism to antagonism depending on host, symbiont genotype, and conditions (Saikkonen et al. 1998; Faeth 2002).

The absence of endophyte-driven shifts in VOC composition contrasts with grass-*Epichloë* systems, in which fungal partners modify host terpenoid outputs under stress (Wang et al. 2020; Realini et al. 2024) and diverges from systems such as peppermint (*Mentha* × *piperita*) where endophyte infection increases overall volatile emission rates (Mucciarelli et al. 2007). The difference likely reflects the distinct spatial organization of the *Oxytropis-Alternaria* symbiosis: *A. oxytropis* resides intercellularly within leaves and reproductive tissues rather than the foliar glands responsible for monoterpene synthesis, and such compartmental separation limits fungal influence on terpene pathways while preserving host control over volatile signaling (Pryor et al. 2009; Oldrup et al. 2010; Noor et al. 2018). *O. sericea* instead aligns more closely with tall fescue (*Lolium arundinaceum*), where *Epichloë* affects volatile emission rate but not composition (Yue et al. 2001).

The one grass system in which a nonanal difference has been reported offers a further comparison. In *Leymus chinensis*, *Epichloë* infection had no effect on constitutive nonanal emission and no significant effect on overall VOC composition, but infected plants emitted substantially more nonanal after locust feeding, an interaction the authors attribute to endophyte-mediated priming of host defense (Deng et al. 2024). Our plants experienced negligible herbivory over a decade of observation at this garden (Keith et al. 2025), so we sampled the constitutive state in which that system also shows no endophyte effect. Whether *A. oxytropis* primes an inducible volatile response in *O. sericea* remains untested, and induction experiments pairing controlled herbivory with headspace collection would be the direct way to ask. The persistence of swainsonine biosynthesis alongside stable terpenoid profiles is consistent with alkaloid production and volatile signaling operating as independent biochemical modules in this system – a decoupling that may reflect ancestral trait retention or a functional integration that only manifests under conditions we did not test.

The evolutionary persistence of vertical transmission despite apparent host neutrality has several non-exclusive explanations. Swainsonine may have conferred defensive benefits under historical selection regimes no longer operating, given that specialist herbivores commonly evolve alkaloid tolerance while livestock sensitivity likely reflects mammalian physiology rather than evolved plant defense. Swainsonine may additionally exert diffuse effects on invertebrate or vertebrate herbivore populations that indirectly benefit infected plants at a population level, a possibility that remains underexplored. Finally, if vertical transmission is highly efficient, selection may favor tolerating a neutral symbiont over investing in exclusion mechanisms – consistent with the finding that endophyte removal conferred no fitness advantage in common garden conditions. Resolving whether this association represents stable commensalism or context-dependent mutualism will require factorial experiments crossing drought, temperature, and nutrient availability, isotopic labeling paired with tissue-specific transcriptomics to quantify host-fungus metabolic fluxes, and multi-generational herbivore trials to test for diffuse alkaloid effects on herbivore populations.

Taken together, our results position the *Oxytropis-Alternaria* association toward the commensal end of the symbiotic continuum, where fungal persistence is maintained by efficient vertical transmission rather than by reciprocal fitness benefits. This conclusion stands in contrast to the mutualistic grass-*Epichloë* systems that have dominated conceptual frameworks for plant-endophyte interactions and suggests that the ecological and evolutionary consequences of endophyte symbiosis are more heterogeneous across plant lineages than those frameworks imply. The *Oxytropis-Alternaria* system offers a tractable model for interrogating the conditions under which endophyte associations stabilize without host benefit – a question with broad relevance to understanding how symbioses originate, persist, and potentially transition along the mutualism-parasitism continuum over evolutionary time.

## Supplementary Information

Supplementary Material 1. Mean volatile organic compound (VOC) emissions (ng g⁻¹ h⁻¹ ± SEM) from whole experimental *Oxytropis* plants grown at a field plot in Bozeman, MT. Compounds are grouped by chemical class. Emissions are compared between plants with and without endophytic fungi. Values represent the average emission per compound from aboveground plant tissues, including leaves and flowers.

Supplementary Material 2. Mean volatile organic compound (VOC) emissions (ng g⁻¹ h⁻¹ ± SEM) from whole experimental *Oxytropis* plants grown in pots in a controlled greenhouse environment. Compounds are grouped by chemical class. Emissions are compared between plants with and without endophytic fungi. Values represent the average emission per compound from aboveground plant tissues, including leaves and flowers.

## Data Availability

The data and code that support the findings of this study are available in a Zenodo repository at https://doi.org/10.5281/zenodo.22928587.
